# Artificial Intelligence Based Pain Assessment Technology in Clinical Application of Real-World Neonatal Blood Sampling

**DOI:** 10.3390/diagnostics12081831

**Published:** 2022-07-29

**Authors:** Xiaoying Cheng, Huaiyu Zhu, Linli Mei, Feixiang Luo, Xiaofei Chen, Yisheng Zhao, Shuohui Chen, Yun Pan

**Affiliations:** 1 Quality Improvement Office, The Children’s Hospital, Zhejiang University School of Medicine, National Clinical Research Center for Child Health, Hangzhou 310052, China; cxynicu@163.com; 2 College of Information Science and Electronic Engineering, Zhejiang University, Hangzhou 310027, China; zhuhuaiyu@zju.edu.cn (H.Z.); zhaoys@zju.edu.cn (Y.Z.); 3 Administration Department of Nosocomial Infection, The Children’s Hospital, Zhejiang University School of Medicine, National Clinical Research Center for Child Health, Hangzhou 310052, China; 22018565@zju.edu.cn; 4 Neonatal Intensive Care Unit, The Children’s Hospital, Zhejiang University School of Medicine, National Clinical Research Center for Child Health, Hangzhou 310052, China; luofeixiang@zju.edu.cn; 5 Gastroenterology Department, The Children’s Hospital, Zhejiang University School of Medicine, National Clinical Research Center for Child Health, Hangzhou 310052, China; hzxiao0914@163.com

**Keywords:** neonatal pain, on-site assessment, artificial intelligence, blood sampling, real-world data

## Abstract

Background: Accurate neonatal pain assessment (NPA) is the key to neonatal pain management, yet it is a challenging task for medical staff. This study aimed to analyze the clinical practicability of the artificial intelligence based NPA (AI-NPA) tool for real-world blood sampling. Method: We performed a prospective study to analyze the consistency of the NPA results given by a self-developed automated NPA system and nurses’ on-site NPAs (OS-NPAs) for 232 newborns during blood sampling in neonatal wards, where the neonatal infant pain scale (NIPS) was used for evaluation. Spearman correlation analysis and the degree of agreement of the pain score and pain grade derived by the NIPS were applied for statistical analysis. Results: Taking the OS-NPA results as the gold standard, the accuracies of the NIPS pain score and pain grade given by the automated NPA system were 88.79% and 95.25%, with kappa values of 0.92 and 0.90 (*p* < 0.001), respectively. Conclusion: The results of the automated NPA system for real-world neonatal blood sampling are highly consistent with the results of the OS-NPA. Considering the great advantages of automated NPA systems in repeatability, efficiency, and cost, it is worth popularizing the AI technique in NPA for precise and efficient neonatal pain management.

## 1. Introduction

Pain assessment and management is one of the research hotspots in the neonatal care field. Although premature and full-term infants usually experience a high frequency of painful stimuli during hospitalization, neonatal pain assessment (NPA) has not been given adequate attention in clinical practice, where painful clinical procedures with a severe degree of pain are prevalent [1]. The operations, such as blood sampling, sputum suction, and indwelling needle puncture, are the major procedures in neonatal care that occur most frequently [2]. These painful procedures are mostly performed on infants within the first 3 days of admission, and no pharmacological interventions are applied for them in most cases [3]. Assessments of continuous pain occurred in less than one-third of neonates, and daily in only 10% [4]. As neonatal pain has a great impact on the short-term and long-term development of newborns [5,6], more attention should be paid to pain management in routine neonatal care.

Accurate NPA is the key to effective pain management. In clinical NPA, the facial expression is considered to be the most explicit indicator, on which a number of pain-assessment scales have been designed, such as the neonatal facial coding system (NFCS) [7], children and infants postoperative pain scale (CHIPPS) [8], premature infant pain profile (PIPP) [9], and neonatal infant pain scale (NIPS) [10]. These NPA scales mainly consider the facial-expression characteristics of newborns, and some of them also integrate factors such as crying, limb movement, and vital signs. Traditional scale-based on-site NPA (OS-NPA) is a process that requires dynamic monitoring by nurses rather than an instantaneous operation. Therefore, regular OS-NPA for pain management is time consuming and laborious. Meanwhile, the results of the OS-NPA could be affected by many factors, including subjective differences in observers [2], interruption from other clinical procedures, a lack of time [11], the gender difference [12], the interference of neonatal activity, inadequate pain-assessment tools [1], and the loss of some transient behaviors.

With the rapid development of artificial intelligence (AI), deep learning has been integrated into neonatal pain-expression-recognition technology [13]. The automatic recognition of neonatal pain expressions has undergone a process from static images to dynamic videos, and from theoretical experiments to system development [14], which has enabled AI-based NPA (AI-NPA). On the one hand, AI-NPA could make up for the shortcomings of the OS-NPA performed by medical staff, and it has the advantages of convenience and efficiency. On the other hand, AI-NPA requires a large number of accurately labeled neonatal pain data to build a model with strong anti-interference ability and high robustness for real-world data.

Unfortunately, the current public reported neonatal pain-expression databases [15,16,17,18] still suffer from a limited number of newborns and samples, deficient population information, limited types of pain stimuli that have large differences with painful clinical procedures, the coarse labeling granularity of pain, etc. Moreover, these state-of-the-art databases and many AI-NPA methods [19,20,21,22] focus on ideal neonatal pain samples, which are samples with restrictions on the neonatal activities and facial posture in the data-collection stage, or with manual screening to avoid disturbed neonatal pain data. These methods make it difficult to meet the clinical requirements regarding accuracy, and they are not feasible for processing neonatal pain data collected in real-world clinical scenes. Hence, there is still a large gap between the current AI-NPA methods and the actual clinical needs in terms of the pain-analysis objectives and scenarios, which has resulted in limited clinical applications.

In order to realize an automated NPA system for actual clinical needs, we previously established a video database of neonatal facial expressions during painful clinical procedures in neonatal wards [23], and we developed an AI-NPA method for real-world data. Our AI-NPA method is robust to facial occlusion and pose variations. Concretely, the method applied generative adversarial networks (GANs) to learn how to recover ideal facial images from real-world facial images with variant poses and occlusion for obtaining modified facial features that enable subsequent interference-adaptive fine-grained neonatal pain assessment. Based on our AI-NPA method, we further completed the design of an automated NPA system for neonatal pain on a mobile platform with an in-hospital server.

In this paper, we propose a prospective study to analyze the consistency of the NPA results given by the automated NPA system and OS-NPA in a real-world clinical operation scenario, thus verifying the clinical practicability of automated NPA systems. A total of 232 newborns who underwent blood-sampling operations in the neonatal wards of the Children’s Hospital of Zhejiang University School of Medicine were recruited and participated in this experiment. Both the OS-NPA performed by two nurses and the AI-NPA given by our automated NPA system were applied during the implementation of four types of blood-sampling operations (i.e., venous, arterial, heel, and fingertip blood sampling) to give their own NPA results in the form of a pain score and pain grade with reference to the NIPS. The correlation and consistency of the OS-NPA and AI-NPA results, as well as the performance of our automated NPA system on real-world clinical neonatal pain data, were derived and analyzed.

The experiment results show that, according to the OS-NPA results on 232 newborns, the accuracy of the automated NPA system was 88.79%, with kappa values of 0.92 and 95.25%, and a kappa value of 0.90 for the NIPS pain score and pain grade, both with *p* < 0.001. This indicates that the NPA results have a high consistency between the on-site evaluation and the AI inference, even in the neonatal pain data in the real-world blood-sampling scenario. This study could provide a performance baseline for the clinical application of automated NPA systems.

## 2. Materials and Methods

### 2.1. Study Design

**Figure 1:**
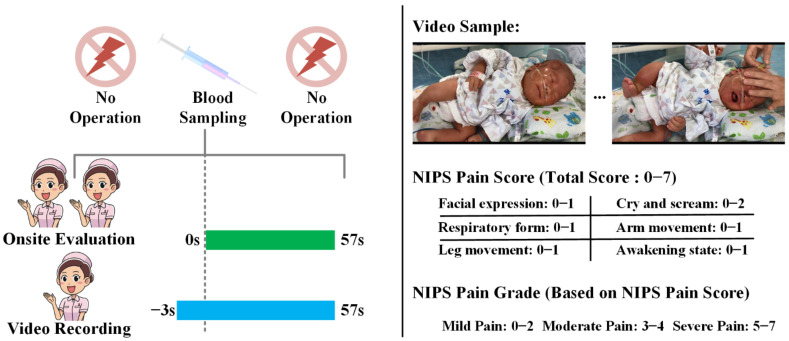
The study design of the clinical practicability of automated NPA systems.

This prospective study was approved by the ethics committee of the Children’s Hospital of Zhejiang University School of Medicine (2018-IRB-051), and parental informed consent was obtained. Newborns who underwent blood-sampling operations in the Department of Neonatology between 1 July 2018 and 30 June 2019 were studied, and the duration of the blood-sampling operations were controlled within 1 min. As shown in Figure 1, when the neonates needed four types of blood sampling (i.e., venous, arterial, heel, and fingertip blood sampling) for clinical diagnosis and treatment, two nurses used the NIPS to perform the respective OS-NPA. Meanwhile, a third nurse used our automated NPA system to shoot the responses of the newborns during the procedure at the bedside, and to retrieve pain scores and pain grades with reference to the NIPS generated by the system. The NIPS pain scores and pain grades of the OS-NPA performed by the two nurses and the AI-NPA performed by the automated NPA system were collected for subsequent statistical analysis.

### 2.2. Inclusion and Exclusion Criteria

Neonates who were hospitalized in the Department of Neonatology for more than 3 days and who underwent the abovementioned four types of blood sampling were included in this prospective study. Exclusion criteria included: serious illness conditions, such as serious birth injury, severe asphyxia, shock, metabolic encephalopathy, moderate and severe hypoxic-ischemic encephalopathy, and severe cardiopulmonary disease; newborns with conditions affecting facial-image acquisition, such as severe congenital malformations, facial malformations, facial nerve injury, and facial surgery. Neonates should not have used sedative or analgesic drugs within 72 h to avoid inaccurate NPA results in the experiment.

### 2.3. OS-NPA Performed by Nurses

Two experienced nurses were assigned to quantitatively assess the pain of the four types of blood sampling for newborns using the NIPS [10]. The pain indicators of the NIPS are defined by the following parameters, with a total score range from 0 to 7 points: facial expression (0–1 point), cry (0–2 points), breathing pattern (0–1 point), position of arms (0–1 point), position of legs (0–1 point), and state of arousal (0–1 point). This is suitable for acute pain assessment, and the pain severity can be classified into mild pain with a score of 0–2, moderate pain with a score of 3–4, and severe pain with a score of 5–7. The Spearman correlation analysis was used to analyze the independent pain-score results of the two nurses, and the correlation coefficient was 0.89 with *p* < 0.001, which indicates the high confidence of the OS-NPA results. Data with a difference in two independent pain scores were further reviewed by these two nurses. They checked the corresponding on-site video to obtain a consistently confirmed pain score as the final OS-NPA result.

### 2.4. AI-NPA Performed by the Automated NPA System

**Figure 2:**
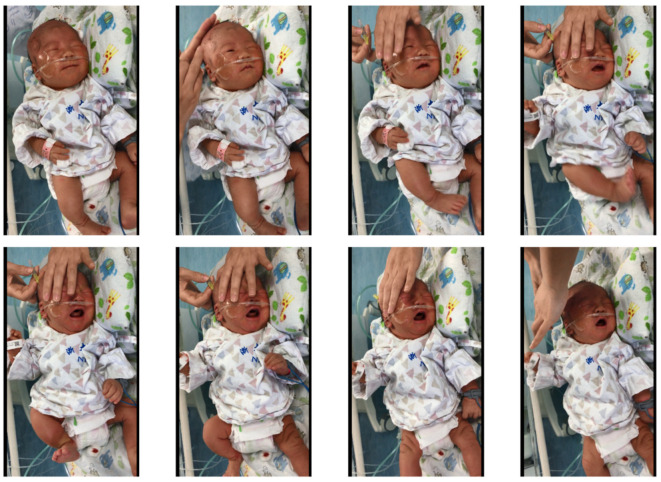
Samples of image sequence in a neonatal pain video.

The automated NPA system was implemented with the client–server model. The application was designed to run on the mobile nursing personal digital assistant (MNPDA) device. When performing the AI-NPA, the nurse could use the application to record videos of the newborns’ behavior during blood sampling. Specifically, the nurse pressed the shooting key 3 s before the blood-sampling operation and the end key 57 s after the operation to achieve a 60 s video for each operation. The heads and faces of the infants were required to be completely presented in the video frame during the whole process. Figure 2 presents the typical image sequence of the neonatal pain video. The video stream was then transmitted to the in-hospital server hosting the AI-NPA model for real-time NPA inference.

The AI-NPA model of our automated NPA system in this study was pretrained on a larger expression-recognition database and was further modulated by a self-built neonatal facial-pain database based on our previous work [23]. Our AI-NPA method includes two parts: unsupervised feature modification and self-attention pain classification. In the unsupervised-feature-modification part, we apply a generative adversarial network (GAN) to modify the facial features toward the direction of face frontalization and deocclusion.

**Figure 3:**
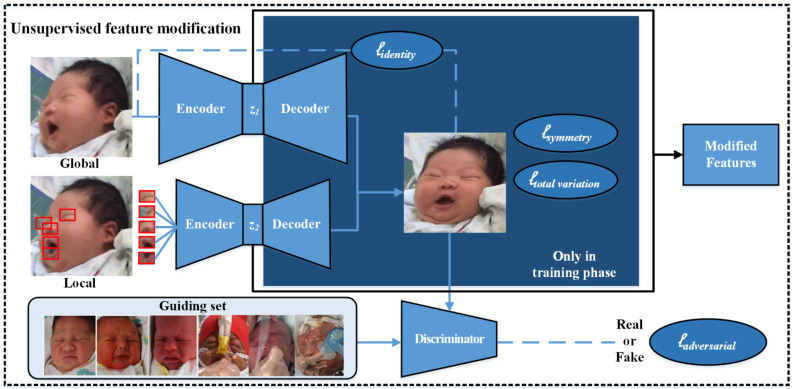
The framework of the unsupervised-feature-modification part in our AI-NPA model.

Specifically, we use the latent vector of the generator as the modified facial features. That is, we only need the encoder network of the generator to extract facial features during the testing phase. The generator of the GAN has two paths, focusing on the reasoning of the global shapes and the transformation of the local details [24]. The discriminator of the GAN is responsible for learning to distinguish between the output of the generator and the normal (frontal and nonoccluded) face images, which pushes the facial features into the manifold of a normal face. We further employed four loss functions: symmetry loss, adversarial loss, identity-preserving loss, and total variation regularization, to train the generator network and discriminator network jointly, as shown in Figure 3.

For the self-attention pain classification, considering that the modified facial features still contain many useless, redundant, and even wrong features, we choose the self-attention mechanism to further filter and enhance the modified features. Specifically, we build an attention branch parallel to the residual branch, which is based on the bottom-up top-down structure [25], and which can output the same size-attention mask that softly weights the facial features.

**Figure 4:**
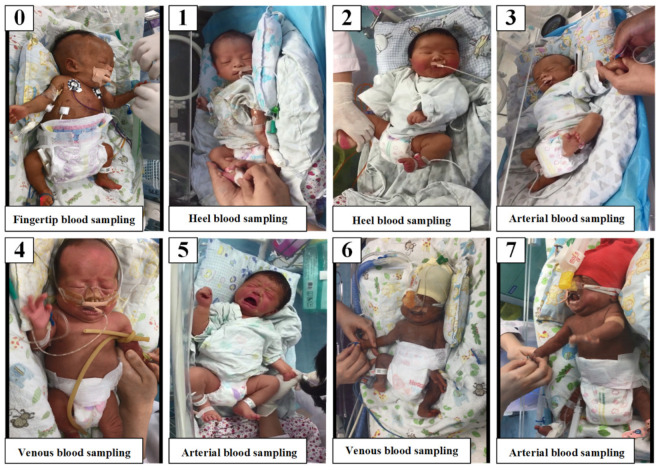
Samples of neonatal pain data with different blood-sampling operations and pain scores.

In the pretraining stage, a total of 12,271 basic expression images in the RAF-DB [26] were applied to obtain the initial model parameters. We then trained this model on 508 neonatal pain images from our real-world neonatal pain database, and we adjusted the parameters to the optimal through the backpropagation algorithm. The samples of our neonatal pain database are shown in Figure 4. The AI-NPA model is deployed on an in-hospital server. The software platform of the server is Ubuntu 16.4.7 with Python 3.6.8, and the hardware configuration is Intel^®^ Xeon^®^ CPU E5-2678 v3 (12/24 cores/threads, 2.5/3.1 GHz base/turbo), 112 GB 2400 MHz DDR4 RAM, and two Nvidia^®^ GeForce^®^ RTX 2080Ti GPUs (1350/1454 MHz base/boost with 11GB GDDR 6 VRAM).

**Figure 5:**
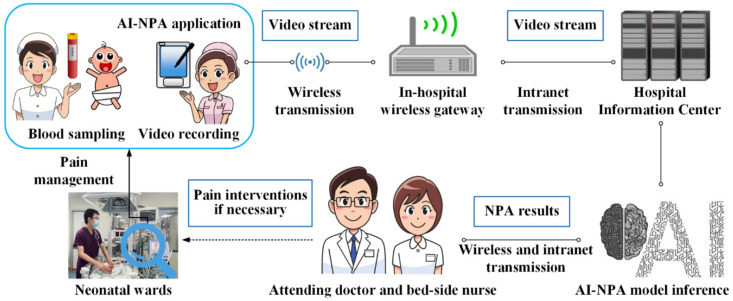
The framework of the automated NPA system and its application.

After the video is transmitted to the server, the AI-NPA model detects the face region of each frame of the image in the video by a built-in algorithm, and it crops the facial image with the size of 224 × 224. Based on the NIPS, the pain scores and pain grades are automatically generated, paired with the images, and stored on the server. The final AI-NPA results are determined as the highest pain score and the corresponding pain grade during one blood-sampling operation, which are used for the subsequent validation of the accuracy of the automated NPA system. The AI-NPA results could be pushed to the application on the MNPDA for on-site nurses or could be checked by the attending doctor for further neonatal pain management, as shown in Figure 5.

### 2.5. Statistical Analysis

Descriptive statistics for the categorical variables were reported as numbers and percentages, and a chi-square test was used for the comparison between groups; statistics were reported as means and standard deviations for continuous variables, and a *t*-test or nonparametric test was used for the group comparison. To analyze the correlation and consistency of the AI-NPA and OS-NPA results, the Spearman correlation analysis, receiver operating characteristic curve (ROC), and kappa coefficient test were used. SAS 9.4 statistical software was used for data analysis, and *p* < 0.05 was considered statistically significant. Because the NIPS pain scores are discrete-integer results, we used the confusion matrix to show the number of cases in which the results were different between the OS-NPA and AI-NPA in detail.

## 3. Results

### 3.1. Study Population

A total of 232 newborns were included in this study, with a mean gestational age of 33.93 ± 4.77 weeks, a mean age of 21 (8.5, 41) days, and a mean birth weight of 2250 ± 1010 g. The detailed demographic characteristics are listed in Table 1. Two nurses performed a total of 464 independent OS-NPAs during 232 neonatal blood-sampling operations for each newborn. As a result, one set of consensus NIPS pain scores and grades was given by the two nurses for every OS-NPA and was used as the gold standard.

### 3.2. Comparison of NIPS Pain Scores between OS-NPA and AI-NPA

The NIPS pain scores of the OS-NPA and AI-NPA were 5.06 ± 1.85 and 5.17 ± 1.84, respectively. The Spearman correlation analysis showed a correlation coefficient of 0.95 (*p* < 0.001) when comparing the NIPS pain scores of the OS-NPA and AI-NPA. The agreement between the two groups was compared, with a kappa value of 0.92 (0.88, 0.95) (*p* < 0.001). The accuracy of the pain score given by the automated NPA system was 88.79% (206/232), and the confusion matrix of the NIPS pain scores for the two methods is shown in Table 2.

### 3.3. Comparison of the NIPS Pain Grades between OS-NPA and AI-NPA

According to the pain-grade criteria of the NIPS, the OS-NPA showed mild pain in 27 patients (8.94%), moderate pain in 47 patients (20.26%), and severe pain in 158 patients (68.10%). The AI-NPA derived mild pain in 26 patients (11.21%), moderate pain in 39 patients (16.81%), and severe pain in 167 patients (71.98%). Compared with the on-site evaluation, the accuracy of the NIPS pain grade given by the AI-NPA was 95.25% (221/232), and the agreement between the two groups was compared, with a kappa value of 0.90 (0.84, 0.96) (*p* < 0.001). See Table 3 for details.

We further investigated the cases of the severe-pain grade. Using the results of the OS-NPA as the criterion, the sensitivity and specificity of the AI-NPA for identifying severe pain were 100% and 87.84%, respectively. The corresponding kappa values and areas under the ROC curve (AUC) of the AI-NPA for identifying severe pain were calculated with different types of blood-sampling operations, as listed in Table 4.

## 4. Discussion

### 4.1. High Evaluation Consistency of the Automated NPA System

The kappa value was used to test the degree of agreement between the results of the different NPA methods. The kappa value ranged from −1 to U+1; the larger the value, the higher the degree of agreement between the two, where the kappa value of the consistency between the NIPS pain scores of the OS-NPA and AI-NPA was 0.92 (0.88, 0.95) (*p* < 0.001). Using the NIPS pain score of the OS-NPA as the gold standard, the accuracy of the pain score given by the automated NPA system was 88.79%. The comparison between the AI-NPA and OS-NPA showed that the correlation coefficient between the two was 0.95 (*p* < 0.001), which was highly positive. These results indicated that there was high consistency in the NIPS pain scores between the AI-NPA and OS-NPA.

Meanwhile, the accuracy of the NIPS pain grade given by the AI-NPA was 95.25%, with a kappa value of 0.90 (0.84, 0.96) (*p* < 0. 001). The AI-NPA was also highly consistent with the OS-NPA regarding the evaluation of the degree of pain. Because the guidelines recommend a stepped approach for neonatal pain management, including environmental, nonpharmacological, and pharmacological interventions, provided that a standardized approach is used to assess the pain [27], in addition to judging the presence or absence of pain, it is also necessary to distinguish the pain grade.

### 4.2. AI-NPA of Severe Pain

The statistical results in Table 4 show that most of the four types of blood-sampling operations in this study were with severe pain, and especially for the venous sampling, of which 88.8% belongs to severe pain. The AI-NPA of severe pain showed an AUC of 0.974, with a 95% confidence interval (0.952, 0.995), and the kappa value was greater than 0.75. The sensitivity and specificity of the AI-NPA for identifying severe pain were 100% and 87.84%, respectively. The relatively low specificity of the AI-NPA of severe pain was due to the fact that the AI assessment mostly focused on the facial expression, while the assessment parameter “crying” of the NIPS varied the most, with corresponding scores of 0–2 assigned to “not crying”, “sobbing”, and “crying”, respectively.

In real-world complex clinical operation scenarios, neonatal crying is easily interfered with by noisy environments, the alarms of monitors, and the occlusion of respiratory-support devices, and there was also an objective difference in the assessment of the “crying” by the nurses. Although the range of scores for “crying” is the largest of all the items in the NIPS, “facial expression” has been proven to be the most specific indicator of pain, and additional information such as “crying” and “physiological signals” could not provide stable performance increments for the AI-NPA methods [28,29,30,31]. The main bottleneck for highly accurate AI-NPAs is still the volume of the current training data.

While it is currently not possible to accurately assess pain scores or pain grades by AI technology, it is feasible to tune an AI model to make a tradeoff between the sensitivity and specificity of pain assessments according to the clinical requirements. Based on the views of our neonatal care experts, we prefer sensitivity so that as many neonates as possible who are suffering from pain can be screened for diagnosis and possible analgesia by physicians. Therefore, the overestimation of severe pain by our AI-NPA method is currently a purposeful choice driven by clinical needs. Although further improvement is required for our AI-NPA method, it is superior to those reported in the relevant literature [19,20,21,22,30,31], and especially in real-world scenarios. Nevertheless, the specificity of the automated NPA system for identifying severe pain in this study was completely acceptable in clinical practice.

### 4.3. Strengths and Limitations of the Automated AI-NPA System

The strengths of our automated AI-NPA system are threefold: It could enable 24 h real-time pain-status monitoring, saving laborious human assessment by medical staff; it is robust to occlusion and extreme facial-pose-change disturbance compared with other automated methods; recorded videos and their automated assessment results in the system could facilitate research and education related to clinical pain assessment.

The main limitation of our automated AI-NPA system is that the videos evaluated by the AI-NPA method are currently recorded by nurses using mobile nursing personal digital assistants. Considering that it is not feasible for medical staff to perform both the medical operation and video filming, the automated NPA system currently requires an additional nurse to record the video. This conflicts with our desire to significantly reduce the workload of medical staff through automated pain assessment, given the fact that healthcare resources are valuable and limited. Therefore, as the AI technology further matures and works with automatic face-tracking algorithms, we will automate the entire process by recording video with bedside cameras in the future.

## 5. Conclusions

Accurate NPA is the premise of standardized neonatal pain management. The AI technique in NPA based on facial recognition can provide convenience for medical staff. This study showed that the automated NPA system could obtain real-time dynamic pain-evaluation results for blood sampling in a real-world neonatal ward scene, which is helpful to implementing the stepped analgesia program for neonatal pain management. Combined with the concept of closed-loop management, the AI technology embedded in the electronic-medical-record (EMR) system in the future will realize the real-time medical intervention for pain by medical staffs. The EMR system can cooperate with the MNPDA to enable bedside nurses to implement care measures in accordance with an automatically generated nursing plan. The downstream health education system can further perform pain-knowledge education for newborns’ family members so as to realize the traceability, standardization, and intelligence of the whole process of pain management.

## Figures and Tables

**Table 1:** Demographic characteristics of newborns in this study.

Metrics		Number of Cases	Composition Ratio (%)
Sex	Male	86	37.07
	Female	146	62.93
Delivery mode	Spontaneous delivery	95	41.13
	Cesarean section	136	58.87
Main diagnosis	Respiratory disorders	113	48.71
	Digestive disorders	63	27.15
	Nervous-system disorders	22	9.48
	Infection disease	34	14.66
Operation type	Venous blood sampling	36	15.52
	Arterial blood sampling	75	32.33
	Heel blood sampling	75	32.33
	Fingertip blood sampling	46	19.82

**Table 2:** Confusion matrix of the NIPS pain scores for OS-NPA and AI-NPA.

OS-NPA	AI-NPA Given by the Automated NPA System							
	0	1	2	3	4	5	6	7
**0**	5	1	0	0	0	0	0	0
**1**	1	5	0	0	0	0	0	0
**2**	0	0	13	1	0	1	0	0
**3**	0	0	0	15	1	1	1	0
**4**	0	0	1	1	21	4	2	0
**5**	0	0	0	0	0	37	2	4
**6**	0	0	0	0	0	2	47	2
**7**	0	0	0	0	0	0	1	63

The background is highlighted for the diagonal of the confusion matrix.

**Table 3:** Consistency analysis of NIPS pain grading by OS-NPA and AI-NPA.

OS-NPA	AI-NPA			Kappa Value and 95%CI	*p* Value
	Mild	Moderate	Severe		
**Mild**	25	1	1	0.90 [0.84, 0.96]	< 0.001
**Moderate**	1	38	8		
**Severe**	0	0	158		

The background is highlighted for the diagonal of the confusion matrix.

**Table 4:** Consistency analysis of AI-NPA and OS-NPA of severe pain.

Operation Type	Number of Subjects with Severe Pain (Proportion %)		Kappa Value and 95% CI	AUC and 95% CI	*p* Value
	OS-NPA	AI-NPA			
Venous blood sampling	32 (88.8)	32 (88.8)	1.00 [1.00, 1.00]	1.000 [1.000, 1.000]	<0.001
Arterial blood sampling	57 (76.0)	60 (80.0)	0.88 [0.76, 1.00]	0.967 [0.924, 1.000]	<0.001
Heel blood sampling	43 (57.3)	46 (61.3)	0.92 [0.83, 1.00]	0.984 [0.959, 1.000]	<0.001
Fingertip blood sampling	26 (56.5)	29 (63.3)	0.86 [0.72, 1.00]	0.957 [0.899, 1.000]	<0.001

## Data Availability

The data are stored at the Children’s Hospital, Zhejiang University School of Medicine, and will be available upon a reasonable request to the corresponding authors with acceptable ethical clearance.
